# Association of the *RPA3-UMAD1* locus with interstitial lung diseases complicated with rheumatoid arthritis in Japanese

**DOI:** 10.1136/annrheumdis-2020-217256

**Published:** 2020-07-31

**Authors:** Yuya Shirai, Suguru Honda, Katsunori Ikari, Masahiro Kanai, Yoshito Takeda, Yoichiro Kamatani, Takayuki Morisaki, Eiichi Tanaka, Atsushi Kumanogoh, Masayoshi Harigai, Yukinori Okada

**Affiliations:** 1 Department of Statistical Genetics, Osaka University Graduate School of Medicine, Suita, Japan; 2 Department of Respiratory Medicine and Clinical Immunology, Osaka University Graduate School of Medicine, Suita City, Japan; 3 Department of Rheumatology, Tokyo Women’s Medical University School of Medicine, Shinjuku-ku, Japan; 4 Department of Biomedical Informatics, Harvard Medical School, Boston, Massachusetts, USA; 5 Laboratory of Complex Trait Genomics, Department of Computational Biology and Medical Sciences, Graduate School of Frontier Sciences, the University of Tokyo, Minato-ku, Japan; 6 Laboratory for Statistical Analysis, RIKEN Center for Integrative Medical Sciences, Yokohama, Japan; 7 Division of Molecular Pathology, The Institute of Medical Science, The University of Tokyo, Minato-ku, Japan; 8 BioBank Japan, The Institute of Medical Science, The University of Tokyo, Minato-ku, Japan; 9 Department of Immunopathology, Immunology Frontier Research Center (WPI-IFReC), Osaka University, Suita, Japan; 10 Integrated Frontier Research for Medical Science Division, Institute for Open and Transdisciplinary Research Initiatives, Osaka University, Suita, Japan; 11 Laboratory of Statistical Immunology, Immunology Frontier Research Center (WPI-IFReC), Osaka University, Suita, Japan

**Keywords:** pulmonary fibrosis, rheumatoid arthritis, gene polymorphism

## Abstract

**Objectives:**

The genetic background of rheumatoid arthritis–interstitial lung disease (RA-ILD) has been evaluated in Europeans, but little knowledge has been obtained in non-Europeans. This study aimed to elucidate genome-wide risk of RA-ILD in non-Europeans.

**Methods:**

We performed an initial genome-wide association study (GWAS) of RA-ILD in the Japanese population. By conducting the meta-analysis of the three GWAS datasets of the RA cohorts and biobank of Japanese, our study included 358 RA-ILD cases and 4550 RA subjects without ILD. We then conducted the stratified analysis of the effect of the GWAS risk allele in each CT image pattern.

**Results:**

We identified one novel RA-ILD risk locus at 7p21 that satisfied the genome-wide significance threshold (rs12702634 at *RPA3-UMAD1*, OR=2.04, 95% CI 1.59 to 2.60, p=1.5×10^−8^). Subsequent stratified analysis based on the CT image patterns demonstrated that the effect size of the RA-ILD risk allele (rs12702634-C) was large with the UIP pattern (OR=1.86, 95% CI 0.97 to 3.58, p=0.062) and the probable UIP pattern (OR=2.26, 95% CI 1.36 to 3.73, p=0.0015).

**Conclusion:**

We revealed one novel genetic association with RA-ILD in Japanese. The RA-ILD risk of the identified variant at *RPA3-UMAD1* was relatively high in the CT image patterns related to fibrosis. Our study should contribute to elucidation of the complicated aetiology of RA-ILD.

**Key messages**

**What is already known about this subject?**

- The genetic background of rheumatoid arthritis–interstitial lung disease (RA-ILD) has been evaluated in Europeans, but little knowledge has been obtained in non-Europeans.
- The genetic background of RA-ILD has not yet been evaluated in the context of genome-wide association study (GWAS) in all populations.

**What does this study add?**

- Our GWAS meta-analysis identified one novel RA-ILD risk variant in Japanese (rs12702634 at *RPA3-UMAD1*).
- The RA-ILD risk of the identified variant was relatively high in usual interstitial pneumonia (UIP) and probable UIP patterns on CT, which are related to fibrosis.

**How might this impact on clinical practice or future developments?**

- The risk variant may contribute to RA-ILD risk by altering *RPA3* expression level. Identifying novel risk locus should contribute to elucidation of the complicated aetiology of RA-ILD.

## Introduction

Rheumatoid arthritis (RA) is a systemic inflammatory disease that causes progressive joint destruction with a prevalence of approximately 0.5%–1.0% in developed countries.^1^ Extra-articular complications are estimated to occur in 40% of patients with RA.^2^ Pulmonary complications are common, especially interstitial lung diseases (ILD) are important issues due to its high morbidity and mortality. Risk of death in patients with RA-ILD has been reported to be 2–10 times higher than in patients with RA without ILD^3^ and several studies have shown that usual interstitial pneumonia (UIP) pattern on CT is an indicator of poor prognosis.^4^ Previous studies have demonstrated that patients with RA-ILD have genetic risk loci shared with familial and idiopathic pulmonary fibrosis (IPF).^5 6^ In particular, the *MUC5B* promoter variant (rs35705950) has been noted as a common risk variant with large effect on both IPF and RA-ILD in Europeans.^6^ However, the frequency of the variant varies heterogeneously among ancestral populations. While its minor allele frequency (MAF) was common in Europeans (=0.107 in 1000 Genomes Project Phase 3; 1KGP3), it was as low as 0.008 in east Asians. Further, the MAF in the patients with IPF was markedly different between Europeans and Japanese (=0.33 and 0.034, respectively).^7^ This observation implies that genetic underpinning of RA-ILD is heterogeneous among populations, and identification of additional risk variants other than *MUC5B* in east Asians should be warranted. Furthermore, previous studies assessed RA-ILD risk of the mutations in specific genes implicated with risk of pulmonary fibrosis through candidate gene approaches. There have been no studies that investigated genome-wide risk of RA-ILD.

In this study, we report an initial genome-wide association study (GWAS) of RA-ILD in the Japanese population. We conducted a meta-analysis of the three GWAS datasets of the RA cohorts and biobank of Japanese, which included 358 RA-ILD cases and 4550 RA subjects without ILD.

## Methods

### Study cohorts and subjects

This study enrolled 1117 and 3899 individuals affected with RA from Institute of Rheumatology Rheumatoid Arthritis (IORRA) and BioBank Japan (BBJ), respectively.^8^ All the subjects fulfilled the 1987 criteria of the American College of Rheumatology for RA diagnosis. IORRA is an RA cohort which was established in 2000 at Tokyo Women’s Medical University, Japan.^9^ All the RA subjects in the IORRA cohort had high-dose CT in a variety of clinical settings. The ILD status of each subject was classified as UIP, probable UIP, non-specific interstitial pneumonia (NSIP), unclassifiable, others and normal according to the international criteria.^10^ In the case of a slight shadow that is considered to be clinically insignificant, it was classified into unclassifiable when we confirmed the respiratory symptoms and the description of ILD on the medical record; otherwise, it was classified into others. Of the CT image patterns, UIP, probable UIP, NSIP and unclassifiable were defined as RA-ILD. The chest CT image of each subject was reviewed by an experienced physician, and findings were confirmed based on the concordance with those by the radiologist’s report. There existed few cases with discrepancies between the physician’s reviews and the radiologist’s reports. In such cases, the physician re-examined the chest CT images again in consideration of the radiologist’s reports. Interstitial pneumonia on CT was further confirmed to be clinically RA-ILD by referring the medical record. BBJ is a patient-based cohort with multiomics data from genotype to multitude phenotype.^11^ The status of RA or ILD in BBJ was examined through interviews and reviews of medical records. While we did not have access to individual CT reports in the BBJ cohort, ILD was comprehensively diagnosed based on various clinical findings including a CT scan in medical practice. All the subjects agreed with informed consent based on the approval of the institutional ethical committee.

### GWAS genotyping and imputation

GWAS genotyping of the subjects were conducted using the Illumina HumanCoreExome (grouped as ‘IORRA1’), Illumina HumanOmniExpress BeadChip (grouped as ‘IORRA2’) in the IORRA cohort,^12^ and the Illumina HumanOmniExpressExome BeadChip or a combination of the Illumina HumanOmniExpress and HumanExome BeadChips in the BBJ cohort.^13^ Quality control of subjects was performed according to the following exclusion criteria: (1) sample call rate <0.98, (2) closely related individuals with PI_HAT>0.125, which was calculated using PLINK, and (3) non-East Asian outliers estimated by principal component analysis using smartpca. Variants satisfying the following criteria were also excluded: (1) single nucleotide polymorphism (SNP) call rate <0.99, (2) MAF <0.01, (3) Hardy-Weinberg equilibrium p-value ≤1.0 × 10^−6^, as described elsewhere.^14^

Haplotype phasing of the GWAS data was performed using SHAPEIT2 in the IORRA cohort and Eagle (V.2.3) in the BBJ cohort. Genotype dosages were imputed using Minimac3 in the BBJ cohort and Minimac4 in the IORRA cohort with the population-specific reference panel of Japanese, which was integrated whole-genome sequence data of 1000 Genomes Project Phase 3 (V.5) and 1037 Japanese.^15^ Variants with imputation quality *Rsq* <0.5 or MAF <0.02 in either case or control were excluded from the subsequent association analysis. The summary of genotyping platforms and quality control criteria for the subjects and variants is described in online supplementary table 1.

10.1136/annrheumdis-2020-217256.supp1 Supplementary data

### RA-ILD GWAS and meta-analysis

In the GWAS, we evaluated association between each variant and RA-ILD by logistic regression implemented in PLINK, assuming additive effect of the allele dosages. Age, sex and the top two principal components were included as covariates in the regression model. Meta-analysis of the GWAS results was performed by an inverse-variance method assuming a fixed-effects model on the effect estimates using METASOFT. METASOFT was also applied to calculate Cochran’s *Q* statistic and to estimate posterior probability that the effect exists in each study (ie, *M-*value).^16^ Briefly, *M*-value is estimated by the Bayesian method from the prior probability, that follows a uniform distribution at the default setting, assuming a model that effect estimates follow a normal distribution whose mean is *μ* (≠0) when the study has effect, and the mean is 0 when it has no effect. *M*=0.9 was adopted as a significance level for effect to exist, as was done in the original study. Genome-wide significance and suggestive thresholds were set at the level of p=5.0 × 10^−8^ and 5.0×10^−6^, respectively. The genomic control factor λ_GC_ was calculated using R statistical software.

### Functional annotation and fine mapping of the candidate gene

Regional plots were created with LocusZoom for the locus where genome-wide significant or the suggestive lead variants were included. Annotation of promotor and enhancer marks for the variants was searched through HaploReg (V.4.1). Quantitative effects on gene expression levels of the variants (ie, eQTL effect) were queried according to GTEx Portal (V.8). Positional candidate genes, where the variants were located or closest, and eGenes, which showed significant eQTL associations with them, were evaluated.

### Patient and public involvement

This research was done without patient and public involvement. Patients and public were not invited to comment on the study design and were not consulted to develop patient relevant outcomes or interpret the results.

## Results

**Figure 1:**
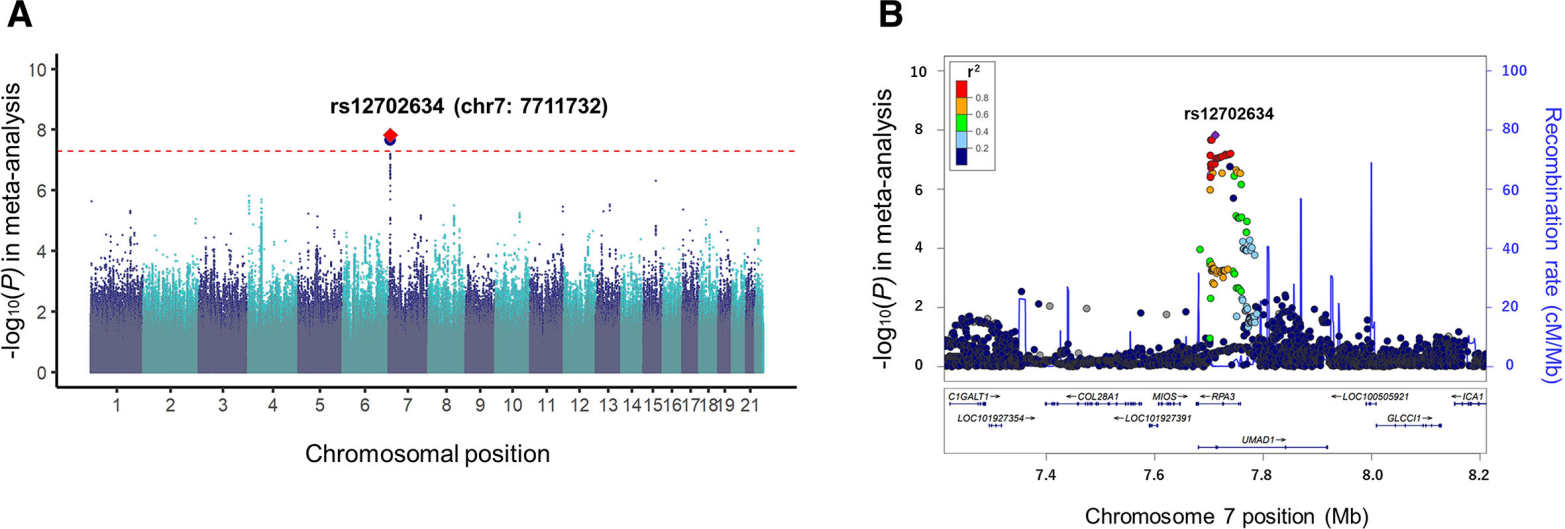
Association results of the genome-wide association study (GWAS) meta-analysis for the three datasets. (A) A Manhattan plot of the GWAS meta-analysis of rheumatoid arthritis–interstitial lung diseases in the Japanese population. The locus which satisfied the genome-wide significance threshold is highlighted. The diamond coloured in red is the lead variant of the locus, which is labelled by rsID and position defined by NCBI build GRCh37. The horizontal dashed red line indicates the genome-wide significance threshold (p=5.0 × 10^−8^). (B) A regional plot of the *RPA3-UMAD1* locus. The lead variant of rs12702634 is coloured in purple and all the other variants are coloured based on linkage disequilibrium (LD) with the lead variant as in the legend. The LD statistics of *r^2^* was calculated using the east Asian (EAS) reference panel of 1000 Genomes Project Phase 3 V.5.

**Figure 2:**
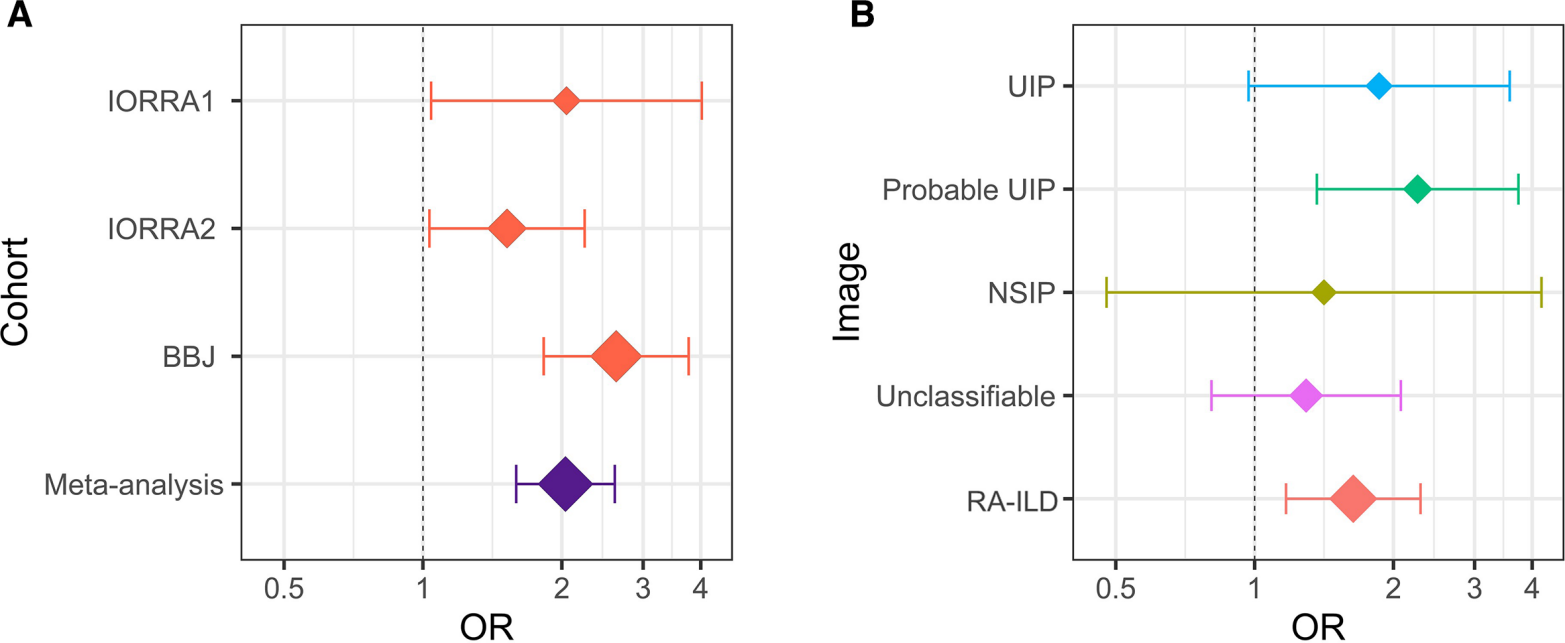
The OR and the 95% CI of rs12702634-C. (A) A forest plot of rs12702634-C in each dataset and meta-analysis. The red diamonds indicate the OR in each dataset and the purple diamond indicates the OR estimated by meta-analysis of the three datasets. (B) A forest plot of rs12702634-C in each CT image pattern. The diamonds indicate the ORs estimated by meta-analysis of the two Institute of Rheumatology Rheumatoid Arthritis (IORRA) datasets (IORRA1 and IORRA2) in each CT image pattern. Rheumatoid arthritis–interstitial lung diseases (RA-ILD) was classified into the four CT image patterns (usual interstitial pneumonia (UIP), probable UIP, non-specific interstitial pneumonia (NSIP) and unclassifiable). The vertical dotted line indicates the OR of 1 and the *x*-axis is drawn with a logarithmic scale in the both figures. The diamond size reflects sample size of the individual categories. The whiskers represent 95% CIs. BBJ, BioBank Japan.

In the GWAS meta-analysis, we integrated three GWAS datasets consisting of IORRA1 (n=236), IORRA2 (n=876) and BBJ (n=3796) after the quality control as described in the Methods section. We tested 5 934 489 variants available in all the three datasets, which consisted of 358 cases (RA-ILD) and 4550 controls (RA without ILD; online supplementary table 1). The genomic control factor did not show apparent inflation (λ_GC_ = 0.98), suggesting no apparent bias in the meta-analysis due to confounding population structure (online supplementary figure 1). In the GWAS meta-analysis results, we identified one novel locus at 7p21 that satisfied the genome-wide significance threshold of p*<*5.0 × 10^−8^ (rs12702634, OR=2.04, 95% CI 1.59 to 2.60, p=1.5 × 10^−8^; figure 1A and table 1). The lead variant of rs12702634 was obtained by genotype imputation with high accuracy (*Rsq*=0.69, 0.90, and 1.00 in IORRA1, IORRA2 and BBJ, respectively). Directional effects of the RA-ILD risk allele of rs12702634-C were consistent among the three GWAS datasets, constantly demonstrating nominal association significance in each dataset (p<0.05; figure 2A and table 1).

**Table 1:** Genome-wide significant variant associated with rheumatoid arthritis–interstitial lung diseases

SNP	Chr	Position (bp)	Candidate gene	eGene	Dataset	No of subjects		REF allele frequency		OR (95% CI)*	P value
						Case	Control	Case	Control		
rs12702634	7	7 711 732	*RPA3* *UMAD1*	*RPA3*	IORRA1	60	176	0.17	0.11	2.05 (1.04 to 4.02)	0.038
					IORRA2	195	681	0.11	0.079	1.52 (1.03 to 2.24)	0.034
					BBJ	103	3693	0.19	0.080	2.62 (1.83 to 3.77)	1.7×10^−7^
					Meta-analysis	358	4550	0.14	0.081	2.04 (1.59 to 2.60)	1.5×10^−8^

*OR of the reference allele (rs12702634-C).

BBJ, BioBank Japan; IORRA, Institute of Rheumatology Rheumatoid Arthritis.

The heterogeneity of the effect size was not apparent (*Q*=4.06, p=0.13). The *M*-values for the variant were above 0.9 among all the three datasets, which indicated that the RA-ILD risk of the allele existed regardless of dataset differences (online supplementary figure 2). The genetic loci with suggestive association signals of p<5.0 × 10^−6^ are listed in online supplementary table 2. We tried to check the association of the *MUC5B* promoter variant (rs35705950), an RA-ILD common risk variant in Europeans,^6^ but we could not assess it because the variant was difficult to be imputed with enough quality due to the rare allele frequency (MAF=0.006 in our population-specific imputation reference panel of Japanese^15^). Results of the previously reported IPF risk loci other than *MUC5B* were indicated in online supplementary table 3.

We then conducted the stratified analysis of the RA-ILD risk of the rs12702634-C allele in each CT image pattern by performing the meta-analysis of the two IORRA datasets, where detailed image patterns were recorded. The OR in the overall RA-ILD was 1.64 (95% CI 1.17 to 2.29, p=0.0041; online supplementary table 4). The OR in the UIP and probable UIP patterns was 1.86 (95% CI 0.97 to 3.58, p=0.062) and 2.26 (95% CI 1.36 to 3.73, p=0.0015), respectively, which were relatively higher than overall RA-ILD and other CT image patterns (figure 2B). On the other hand, the OR in the NSIP and unclassifiable patterns was relatively low (=1.41 and 1.29, respectively). These results suggested that the RA-ILD risk of rs12702634-C was heterogeneous according to the CT image patterns, being high risk in the probable UIP and UIP patterns.

We performed the analysis by confining the subjects to who had high-resolution CT (HRCT) in the IORRA cohort (n=1004 out of 1112). Although the overall statistical significance slightly decreased, association of rs12702634 with RA-ILD was still significant in the meta-analysis of the three datasets (OR=2.03, 95% CI 1.58 to 2.60, p=2.5 × 10^−8^; online supplementary table 5). In the stratified analysis based on the HRCT image patterns, we obtained similar results to those of CT (online supplementary table 6). In the large-scale GWAS of Japanese, rs12702634-C demonstrated nominally significant increased risk of ILD in general populations (OR=1.25, 95% CI 1.05 to 1.49, p=0.013; online supplementary table 7).^17^ No association with any smoking behaviours was observed (p>0.18).^18^

The RA-ILD risk variant of rs12702634 was located in the intron region of *RPA3* and *UMAD1* (figure 1B). This variant was located within the enhancer histone marks in several immune cells, indicating potential involvements is gene expression profile modulations in the immune system. Of note, the GTEx data showed that the rs12702634-C risk allele increased *RPA3* expression levels in various tissues including lung (online supplementary figure 3).

## Discussion

In this study, we conducted an initial GWAS meta-analysis of RA-ILD, which identified *RPA3-UMAD1* at 7p21 as a novel risk locus in the Japanese population. The lead variant of rs12702634 has been reported as an eQTL that affects *RPA3* expression levels, implying *RPA3* as a potential risk gene in the region. According to the recent single cell RNA-seq research,^19^ *RPA3* is most abundantly transcribed in blood cells such as erythroid cells and proliferating T cells, where cell-specific functional roles of the risk variant should be elucidated. RPA3 is a part of the heterotrimeric replication protein A complex (RPA), which plays an essential role in DNA replication and the cellular response to DNA damage. RPA has also been reported to regulate telomere elongation through modulation of telomerase activity.^20^ Telomere dysfunction induces DNA damage, triggers senescence and causes adverse effects on various organs, and pulmonary fibrosis is one of the most common manifestations of telomere-mediated disease.^21^ In addition, telomere lengths in RA-ILD have been reported to be shorter in patients with RA-ILD a *TERT*, *RTEL1* or *PARN* mutation compared with healthy individuals.^5^ In our study, the IPF risk variant of rs2736100 in *TERT* demonstrated nominally significant association with RA-ILD (p=4.0 × 10^−4^; online supplementary table 3). Considering these observations, further studies examining functional roles of the *RPA3* variants including rs12702634 in alteration of telomere function should be warranted.

The stratified analysis by the CT image patterns showed that the RA-ILD risk of rs12702634-C was relatively high in the UIP and probable UIP patterns. Previous studies have reported that patients with interstitial pneumonia in the probable UIP pattern on CT are highly likely to show the UIP pattern on histological examination.^22^ The main histopathological features of the UIP pattern is interstitial fibrosis with spatial heterogeneity. The RA-ILD risk of rs12702634-C was also nominally associated with ILD risk in general Japanese populations.^17^ Considering these observations, the functional role of rs12702634 might be involved in the aetiology of fibrosis. Fibrosis leads to irreversible destruction of alveolar structures, resulting in reduced respiratory function in patients with RA. Elucidation of pathological mechanisms of fibrosis provides new opportunity for protecting respiratory function.

In conclusion, our GWAS meta-analysis identified one novel risk locus associated with RA-ILD in Japanese. The RA-ILD risk of the identified variant at *RPA3-UMAD1* was relatively high in the CT image patterns related to fibrosis. Our study provides novel insights to genetic background and aetiology of RA-ILD, which should contribute to implementation of personalised medicine of patients with RA.
