# Supplementary material for: Association of the RPA3-UMAD1 locus with interstitial lung diseases complicated with rheumatoid arthritis in Japanese
Source: Ann Rheum Dis. 2020 Jul 31;79(10):1305–9. doi: 10.1136/annrheumdis-2020-217256 (PMC7509520; doi:10.1136/annrheumdis-2020-217256)
Supplement: Supplementary data [file annrheumdis-2020-217256supp001.pdf]

Association of the *RPA3-UMAD1* locus with interstitial lung diseases complicated with rheumatoid arthritis in Japanese.

Shirai Y et al.

Shirai Y, *et al. Ann Rheum Dis* 2020; 79:1305–1309. doi: 10.1136/annrheumdis-2020-217256

Supplementary Table 1. Characteristics of the three datasets.

Dataset	No. case	No. control	Genotyping platform	Pre-impuation QC criteria							Post-impuation QC criteria		
				Sample call rate	PI_HAT	SNP call rate	MAF	HWE p-value	No. excluded subjects	No. excluded variants	MAF	*Rsq*	No. variants after QC
IORRA1	60	176	Illumina HumanCoreExome	> 0.98	< 0.125	> 0.99	> 0.01	$> 1.0\times10^{-6}$	5	2,064	> 0.02	> 0.5	6,139,219
IORRA2	195	681	Illumina HumanOmniExpress BeadChip	> 0.98	< 0.125	> 0.99	> 0.01	$> 1.0\times10^{-6}$	0	5,118	> 0.02	> 0.5	6,567,125
BBJ	103	3,693	HumanExome BeadChip<br>Illumina HumanOmniExpress BeadChip<br>Illumina HumanOmniExpressExome BeadChip	> 0.98	< 0.125	> 0.99	> 0.01	$> 1.0\times10^{-6}$	103	13,701	> 0.02	> 0.5	6,510,436
Meta-analysis	358	4,550	-	-		-	-	-	108	-	-	-	5,934,489

Supplementary Table 2. Suggestive variants ($P < 5.0 \times 10^{-6}$) associated with RA-ILD.

SNP	Chr	Position (bp)	Candidate gene	eGene	Alleles REF	Alleles ALT	Dataset	Minor allele freq. Case	Minor allele freq. Control	OR [95%CI]*	*P*
rs531367212	1	4,149,083	*EEF1DP6*	-	A	T	IORRA1	0.073	0.035	3.15 [0.99-10.0]	0.052
							IORRA2	0.043	0.025	2.68 [1.24-5.80]	0.013
							BBJ	0.068	0.032	3.29 [1.70-6.38]	4.1×10^{-4}
							Meta-analysis	0.055	0.031	3.04 [1.92-4.82]	2.3×10^{-6}
rs732814	1	201,689,398	*NAV1, IPO9-AS1*	*NAV1*	A	G	IORRA1	0.14	0.21	0.54 [0.29-1.01]	0.053
							IORRA2	0.16	0.22	0.60 [0.44-0.82]	1.3×10^{-3}
							BBJ	0.12	0.2	0.56 [0.36-0.86]	8.1×10^{-3}
							Meta-analysis	0.14	0.2	0.58 [0.46-0.73]	4.7×10^{-6}
rs6842024	4	4,577,212	*STX18-AS1*	*STX18*	G	C	IORRA1	0.42	0.29	1.88 [1.19-2.96]	7.0×10^{-3}
							IORRA2	0.42	0.34	1.39 [1.10-1.76]	5.7×10^{-3}
							BBJ	0.45	0.36	1.56 [1.18-2.07]	1.9×10^{-3}
							Meta-analysis	0.43	0.35	1.51 [1.28-1.78]	1.5×10^{-6}
rs9291340	4	49,178,135	*AC118282.3*	-	T	C	IORRA1	0.35	0.26	2.37 [1.24-4.54]	9.1×10^{-3}
							IORRA2	0.38	0.31	1.54 [1.15-2.05]	3.4×10^{-3}
							BBJ	0.41	0.32	1.64 [1.18-2.28]	3.3×10^{-3}
							Meta-analysis	0.38	0.32	1.65 [1.34-2.02]	2.0×10^{-6}
rs79857884	7	5,908,436	*RN7SL556P*	-	A	G	IORRA1	0.022	0.025	0.99 [0.17-5.96]	0.99
							IORRA2	0.039	0.026	1.63 [0.85-3.14]	0.14
							BBJ	0.095	0.03	5.04 [2.89-8.77]	1.1×10^{-8}
							Meta-analysis	0.053	0.029	2.96 [1.96-4.46]	2.3×10^{-7}
rs199739054	8	101,169,823	*AP003469.4*	-	A	AAT	IORRA1	0.11	0.082	1.50 [0.72-3.12]	0.28
							IORRA2	0.14	0.1	1.57 [1.10-2.24]	0.013
							BBJ	0.19	0.091	2.17 [1.49-3.16]	5.7×10^{-5}
							Meta-analysis	0.15	0.092	1.79[1.40-2.28]	3.1×10^{-6}
rs76140786	11	131,512,017	*NTM, AP003025.1*	-	G	T	IORRA1	0.18	0.15	1.46 [0.71-3.01]	0.31
							IORRA2	0.22	0.14	2.01 [1.45-2.79]	3.2×10^{-5}
							BBJ	0.2	0.15	1.53 [1.04-2.26]	0.032
							Meta-analysis	0.21	0.14	1.75 [1.38-2.23]	3.4×10^{-6}
rs17062507	13	74,872,156	*RIOK3P1*	-	G	A	IORRA1	0.089	0.063	1.86 [0.79-4.36]	0.16
							IORRA2	0.11	0.068	1.85 [1.24-2.77]	2.7×10^{-3}
							BBJ	0.13	0.07	2.03 [1.33-3.08]	9.1×10^{-4}
							Meta-analysis	0.11	0.069	1.93 [1.46-2.54]	2.9×10^{-6}
rs57947079	15	78,716,386	*CHRNB4, AC022748.2*	-	C	T	IORRA1	0.25	0.19	1.50 [0.81-2.75]	0.19
							IORRA2	0.26	0.17	1.87 [1.41-2.47]	1.2×10^{-5}
							BBJ	0.25	0.19	1.49 [1.07-2.08]	0.018
							Meta-analysis	0.26	0.18	1.68 [1.37-2.05]	4.8×10^{-7}
rs9906052	17	2,691,793	*CLUH*	-	T	C	IORRA1	0.2	0.16	1.72 [0.84-3.50]	0.14
							IORRA2	0.22	0.17	1.48 [1.09-2.01]	0.012
							BBJ	0.27	0.18	1.93 [1.37-2.73]	1.9×10^{-4}
							Meta-analysis	0.23	0.18	1.67 [1.34-2.08]	4.3×10^{-6}

*Odds ratio of minor allele to major allele.

Shirai Y, *et al. Ann Rheum Dis* 2020; 79:1305–1309. doi: 10.1136/annrheumdis-2020-217256

Supplementary Table 3. Summary statistics of the reported IPF risk loci in the meta-analysis.

SNP	Chr	Position (bp)	Gene	Alleles		OR [95%CI]*	*P*
				REF	ALT		
rs78238620	3	44,902,386	*KIF15*	T	A	1.59 [1.1-2.28]	0.013
rs6793295	3	169,518,455	*LRRC34*	T	C	0.96 [0.79-1.17]	0.69
rs2609255	4	89,811,195	*FAM13A*	G	T	1.12 [0.95-1.32]	0.19
rs2736100	5	1,286,516	*TERT*	C	A	0.73 [0.61-0.87]	4.0×10^{-4}
rs2076295	6	7,563,232	*DSP*	T	G	1.22 [1.04-1.43]	0.018
rs7887	6	31,864,547	*EHMT2*	G	T	1.04 [0.86-1.26]	0.67
rs12699415	7	1,909,479	*MAD1L1*	A	G	1.05 [0.89-1.24]	0.55
rs4727443	7	99,593,346	*RP4-604G5.3*	C	A	0.84 [0.71-0.99]	0.043
rs28513081	8	120,934,126	*DEPTOR*	A	G	0.78 [0.55-1.11]	0.16
rs11191865	10	105,672,842	*OBFC1*	G	A	0.92 [0.77-1.09]	0.32
rs1278769	13	113,536,627	*ATP11A*	A	G	0.93 [0.78-1.11]	0.42
rs2034650	15	40,717,302	*IVD*	G	A	0.83 [0.65-1.07]	0.15
rs17690703	17	43,925,297	*SPPL2C*	C	T	0.99 [0.67-1.46]	0.95
rs12610495	19	4,717,672	*DPP9*	A	G	1.13 [0.89-1.43]	0.32

*Odds ratio of minor allele to major allele.

Shirai Y, *et al. Ann Rheum Dis* 2020; 79:1305–1309. doi: 10.1136/annrheumdis-2020-217256

Supplementary Table 4. Summary statistics of rs12702634 in the meta-analysis of the two IORRA datasets for each image pattern.

Image pattern	IORRA1 & IORRA2					
	No. subjects		REF allele frequency		OR [95%CI]*	*P*
	Case	Control	Case	Control		
UIP	44	857	0.14	0.084	1.86 [0.97-3.58]	0.062
Probable UIP	68	857	0.16	0.084	2.26 [1.36-3.73]	0.0015
NSIP	20	857	0.11	0.084	1.41 [0.48-4.19]	0.53
Unclassifiable	123	857	0.10	0.084	1.29 [0.81-2.08]	0.29
RA-ILD	255	857	0.13	0.084	1.64 [1.17-2.29]	0.0041

*Odds ratio of the reference allele (rs12702634-C).

Shirai Y, *et al. Ann Rheum Dis* 2020; 79:1305–1309. doi: 10.1136/annrheumdis-2020-217256

Supplementary Table 5. Meta-analysis for the RA subjects in the BBJ cohort and the RA subjects who had HRCT in the IORRA cohort.

SNP	Chr	Position (bp)	Candidategene	eGene	Alleles		Cohort	No. subjects		REF allele frequency		OR (95%CI)*	*P*
					REF	ALT		Case	Control	Case	Control		
rs12702634	7	7,711,732	*RPA3, UMAD1*	*RPA3*	C	G	IORRA1	58	160	0.17	0.11	1.98 [1.03-3.81]	0.051
							IORRA2	178	608	0.1	0.08	1.49 [1.00-2.22]	0.042
							BBJ	103	3,693	0.19	0.08	2.62 [1.83-3.77]	1.7×10^{-7}
							Meta-analysis	339	4,461	0.15	0.08	2.03 [1.58-2.60]	2.5×10^{-8}

*Odds ratio of the reference allele (rs12702634-C).

Shirai Y, *et al. Ann Rheum Dis* 2020; 79:1305–1309. doi: 10.1136/annrheumdis-2020-217256

Supplementary Table 6. Summary statistics of rs12702634 in the meta-analysis of the two IORRA datasets for each image pattern with HRCT.

Image pattern	IORRA1 & IORRA2					
	No. subjects		REF allele frequency		OR [95%CI]*	*P*
	Case	Control	Case	Control		
UIP	39	768	0.13	0.085	1.69 [0.83-3.43]	0.15
Probable UIP	62	768	0.16	0.085	2.23 [1.31-3.77]	0.0029
NSIP	20	768	0.11	0.085	1.41 [0.48-4.15]	0.54
Unclassifiable	115	768	0.11	0.085	1.38 [0.85-2.22]	0.19
RA-ILD	236	768	0.13	0.085	1.61 [1.14-2.26]	0.0063

*Odds ratio of the reference allele (rs12702634-C).

Shirai Y, *et al. Ann Rheum Dis* 2020; 79:1305–1309. doi: 10.1136/annrheumdis-2020-217256

Supplementary Table 7. Summary statistics of rs12702634 in other traits of BBJ.

Trait	No. subjects			REF allele frequency			OR [95%CI]*	*P*
	Case	Control	Total	Case	Control	Total		
IP[a]	806	211,647	212,453	0.10	0.087	0.087	1.25 [1.05-1.49]	0.013
Age of smoking initiation[b]	-	-	30,418	-	-	0.089	1.01 [0.98-1.04]	0.65
Cigarettes per day[b]	-	-	72,655	-	-	0.087	1.01 [0.99-1.03]	0.49
Smoking initiation[b]	83,810	81,626	165,436	0.087	0.087	0.087	1.00 [1.00-1.01]	0.71
Smoking cessation[b]	43,163	32,884	76,047	0.088	0.087	0.087	1.01 [1.00-1.01]	0.18

*Odds ratio of minor allele to major allele.

[a] Ishigaki K, et al. ***Nat Genet*** 2020 (Reference 17).
[b] Matoba N, et al. ***Nat Hum Behav*** 2019 (Reference 18).

Shirai Y, *et al. Ann Rheum Dis* 2020; 79:1305–1309. doi: 10.1136/annrheumdis-2020-217256

Supplementary Figure 1. A quantile–quantile (QQ) plot of GWAS meta-analysis.

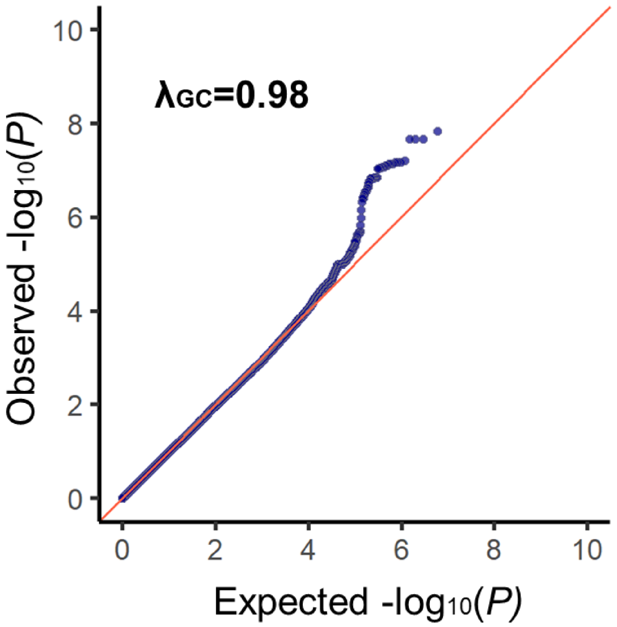

The *x*-axis indicates the expected -$\log_{10}$(*P*-values) and the *y*-axis indicates the observed -$\log_{10}$(*P*-values). This QQ plot shows that GWAS statistics are not apparently inflated as the genomic control factor indicates (λ_{GC} = 0.98).

Shirai Y, *et al. Ann Rheum Dis* 2020; 79:1305–1309. doi: 10.1136/annrheumdis-2020-217256

Supplementary Figure 2. A P–M plot of rs12702634 in each of the three datasets.

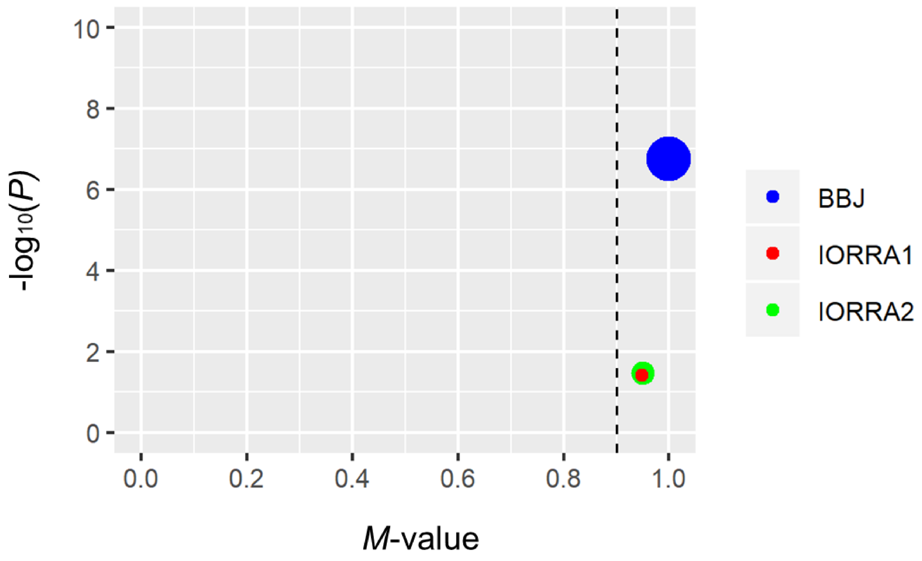

The *x*-axis indicates the *M*-values and the *y*-axis indicates the $-\log_{10}$(*P*-values). The vertical dotted line indicates the significance threshold which has been commonly adopted (*M* = 0.9). The dots are color-coded by individual datasets and their size reflects sample size of the datasets.

Shirai Y, *et al. Ann Rheum Dis* 2020; 79:1305–1309. doi: 10.1136/annrheumdis-2020-217256

Supplementary Figure 3. eQTL effect of rs12702634 on *RPA3* from GTEx portal(ver8).

a **b**

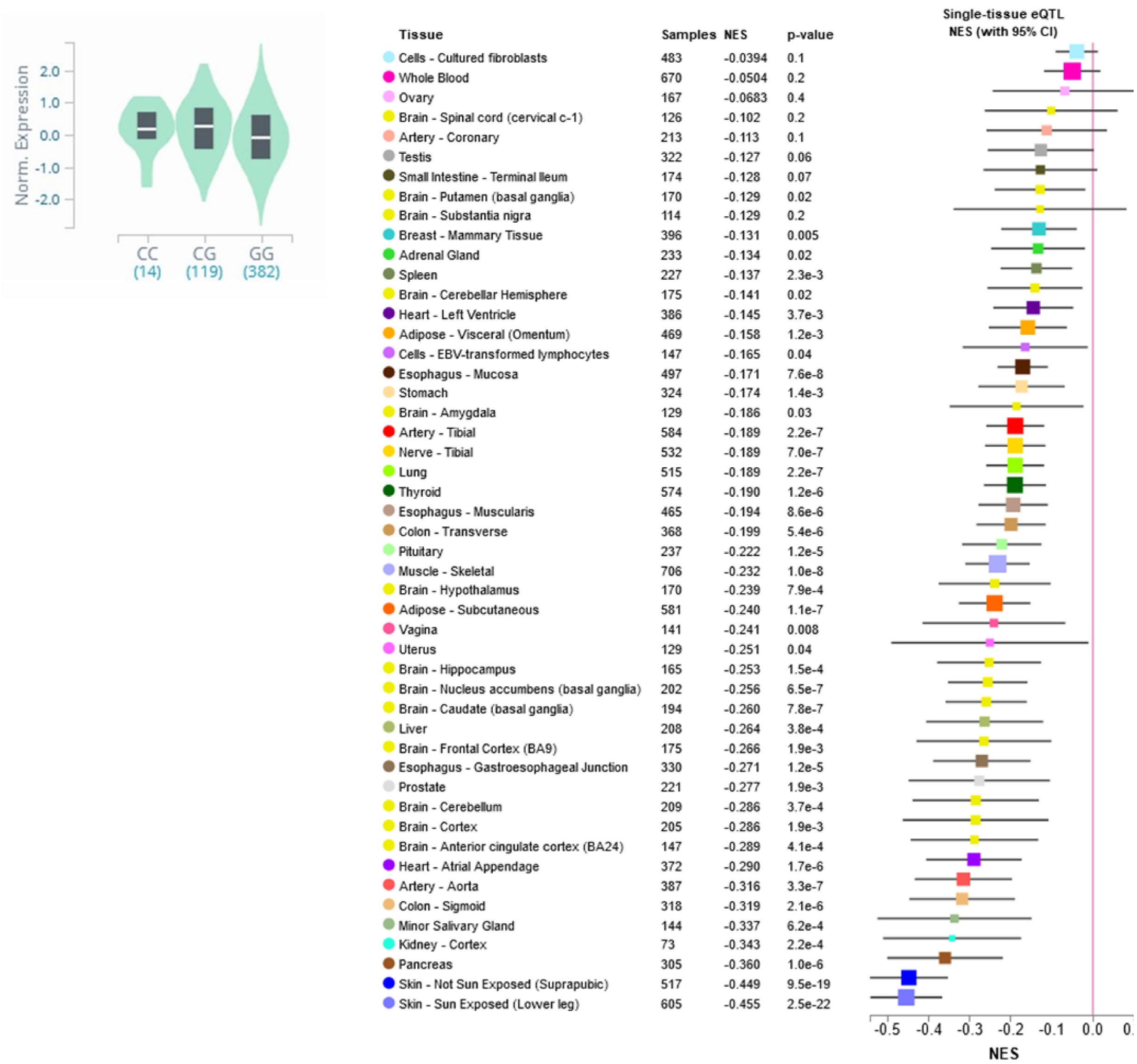

(**a**) A violin plot of *RPA3* expression levels in lung tissue by genotype. The *x*-axis indicates the genotype, where the reference allele is C and the alternate is G. The numbers in the parentheses indicate sample size. The *y*-axis indicates the normalized gene expression level of *RPA3*. (**b**) A box plot of the effect size in multi-tissues. The *x*-axis indicates the normalized effect size (NES) of the alternate allele G of rs12702834. The vertical red line indicates NES of 0. The boxes are color-coded by the tissues and their size reflect sample size of the individual tissues. The whiskers represent 95% confidence intervals.

Shirai Y, *et al. Ann Rheum Dis* 2020; 79:1305–1309. doi: 10.1136/annrheumdis-2020-217256
